# Resistance to the Insulin and Elevated Level of Androgen: A Major Cause of Polycystic Ovary Syndrome

**DOI:** 10.3389/fendo.2021.741764

**Published:** 2021-10-20

**Authors:** Haigang Ding, Juan Zhang, Feng Zhang, Songou Zhang, Xiaozhen Chen, Wenqing Liang, Qiong Xie

**Affiliations:** ^1^ Department of Gynecology, Shaoxing Maternity and Child Health Care Hospital, Shaoxing, China; ^2^ Obstetrics and Gynecology Hospital of Shaoxing University, Shaoxing, China; ^3^ College of Medicine, Shaoxing University, Shaoxing, China; ^4^ Medical Research Center, Zhoushan Hospital of Traditional Chinese Medicine Affiliated to Zhejiang Chinese Medical University, Zhoushan, China; ^5^ Department of Gynecology, Zhoushan Hospital of Traditional Chinese Medicine Affiliated to Zhejiang Chinese Medical University, Zhoushan, China

**Keywords:** polycystic ovary syndrome (PCOS), insulin resistance, androgen, hyper-androgenemia, endocrine disorder

## Abstract

PCOS has a wide range of negative impacts on women’s health and is one of the most frequent reproductive systemic endocrine disorders. PCOS has complex characteristics and symptom heterogeneity due to the several pathways that are involved in the infection and the absence of a comm14on cause. A recent study has shown that the main etiology and endocrine aspects of PCOS are the increased level of androgen, which is also known as “hyperandrogenemia (HA)” and secondly the “insulin resistance (IR)”. The major underlying cause of the polycystic ovary is these two IR and HA, by initiating the disease and its severity or duration. As a consequence, study on Pathogenesis is crucial to understand the effect of “HA” and “IR” on the pathophysiology of numerous symptoms linked to PCOS. A deep understanding of the pattern of the growth in PCOS for HA and IR can help ameliorate the condition, along with adjustments in nutrition and life, as well as the discovery of new medicinal products. However, further research is required to clarify the mutual role of IR and HA on PCOS development.

## Introduction

PCOS, depending on the term employed, is the most predominant endocrine of reproductive women affecting 6-22% of all women globally (1). PCOS has been defined as the present at least two out of the following criteria since the Rotterdam Convention was set up in 2003: clinical or biochemical hyperandrogenism (HA); oligo- or amenorrhea (OM); or ovarian morphological polycystic (PCOM) (2). Along with these three basic characteristics of PCOS, many women also experience many additional comorbidities or concomitant conditions, including insulin resistance (IR) (3), which increases their chance to develop Mellitus diabetes, low-level inflammation, dyslipidemia, and obesity (4, 5).

This definition generates numerous different phenotypes, including phenotypes A (HA, OM, PCOM), B (HA, OM), C (HA, PCOM), and D (HA, PCOM). This is the definition that creates different phenotypes (OM, PCOM). Numerous studies have shown a higher prevalence of PCOS, IR, and other comorbidities among HA (A, B, and C) females, while D was associated with a milder form of PCOS (6). Although two or more women who match PCOS criteria are certainly identified, women who meet only one criterion are often neglected or lost to follow-up because their classification does not comply with PCOS. While it is found that HA without PCOM or OM is harmful to a range of cardiovascular risk factors, women are often left without diagnosis or treatment). While these women may not be experiencing “monthly abnormalities or fertility problems”, “their risk of type 2 diabetes”, “obesity”, “hypertension”, “dyslipidemia”, “metabolic syndrome”, “cardiovascular events” such as myocardial inflammation or stroke may increase with HA-related metabolic hazard (7).

## Polycystic Ovary Syndrome and the Ovarian Cycle

Since no PCOS is known, the most frequently accepted models are multifactorial, in which interactions between environmental and individual features lead to the development of hyperandrogenemia, the biochemical hallmark of the disease. The main cause of most PCOS clinical symptoms is this alteration (8). During the ovary cycle and folliculogenesis, PCOS inhibits several physiological processes. The first phases of folliculogenesis are impaired with increased amounts of Anti-Müllerian Hormones (AMH) (9). AMH is a TGF family of 560 amino acid peptides released by granulosa (GC). It is mainly rich in small antral follicles and has a considerable inhibitory effect on the beginning of primordial follicles (FSH). The AMH levels decline with the follicle, and it appears that low levels for the development from the primordial to the principal phase, prevailing follicle selection, and ovulation of this hormone are required (10). The long-term disorder of ovarian physiology in women with PCOS appears to be significantly influenced by elevated levels of AMH (11) and by poor reproductive outcomes associated with higher levels of AMH (12). PCOS also has the character trait of hypothalamus-hypophysis-ovary axis (HHOA) dysregulation, with increased frequency and amplitude of pulsatile GnRH and luteinized hormone (LH) releasing hormones. More androgen synthesis in the ovarian theca cell (TC) has increased levels of that hormone (13). On the other hand, hyperandrogenemia lowers the sensitivity to estradiol and progesterone of gonadotropic hypothalamic cells, reinforcing the GnRH and LH hypersecretion (14). It is the first of several self-reinforcing pathophysiological cycles in which the development and advancement of PCOS and the existence of symptoms are dependent on hyperandrogenism. Due to the constant proliferation of follicles and the absence of a dominating unit, many of these structures are overstimulated and so retain all of the characteristic hormonal abnormalities, leading to the alternative proposed title ‘polyfollicular ovarian syndrome’ (15). Genetic factors could potentially contribute by predisposing ovarian tissue to the development of this condition in excessively high production of androgen. A probable Mendelian pattern inherited in critical genetic defects, though very variability in penetration based on a variety of environmental and epigenetic factors, such as exposures to higher levels of androgen, is assumed to be the most widely accepted model (16). The model is most commonly accepted. The predisposal to hyperandrogenemia can all be affected by both mutations in androgen receiver, sex hormone-binding globulin (SHBG), and steroidogenic enzymes genes (17).

## Clinical View of the PCOS

In the clinical setting, PCOS manifests itself in a highly varied manner, with a wide range of clinical manifestations (**Table 1**) presents several sets of diagnostic criteria for PCOS (25). While oligomenorrhea is indicative of ovulatory failure, apparent eumenorrhea does not rule out anovulation altogether. Progesterone values of 3-4 ng/mL on days 20–24 of the menstrual cycle is adequate to diagnose an oligo/anovulatory cycle. In contrast, a patient can be classified as anovulatory if at least two following cycles demonstrate anovulation in the presence of hypoprogesteronemia (31). Although IR has not been used as a diagnostic criterion for PCOS in the past, the presence of this change or Acanthosis nigricans in conjunction with hyperandrogenic symptoms is strongly predictive of this syndrome (32). Obesity, like IR, is a common complication in women with PCOS. Nonetheless, these are not always co-occurring, and they may exist independently, resulting in diverse metabolic profiles. Each of these phenotypes exhibits distinct biochemical characteristics that result in distinct risk profiles for cardiovascular disease and fertility (33). Because androgen activity is primarily directed towards the skin, various dermatologic changes associated with hyperandrogenemia can be observed in PCOS, including hirsutism, androgenic alopecia, and acne, as well as seborrhea, onycholysis, and onychorrhexis (34).

**Table 1:** Summary of the PCOS diagnostic characteristics.

Manifestation	Sample size	Epidemiology	Reference Source
Manifestations of hyperandrogenism			
Hirsutism	73	83.8%	(18)
	365	73.2%	(19)
	30	73%	(20)
Acne	30	63%	(20)
	365	49.6%	(19)
Alopecia	70	16%	(21)
Seborrhea	115	34.8%	(22)
Manifestations of ovarian dysfunction			
Oligomenorrhea	30	20%	(20)
	412	74%	(23)
Amenorrhea	365	21.5%	(19)
	30	43%	(20)
Ultrasound polycystic ovaries	412	89%	(23)
	365	97.3%	(19)
Condition			
Obesity	394	80%	(24)
	267	42%	(25)
Insulin resistance	200	71%	(26)
Impaired fasting glucose	254	31.1%	(27)
Type 2 diabetes mellitus	394	6.6%	(24)
Arterial hypertension	346	9%	(28)
Dyslipidemia	200	46.3%	(26)
Metabolic syndrome	129	47.3%	(29)
Mood disorders	103	21%	(30)
	30 53%	30 53%	(20)

## Increased Level of Insulin (Hyperinsulinemia), Insulin Resistance, and Hyper-Androgenemia (Vicious Cycle)

It is commonly established that the participation of IR and hyperinsulinemia in the development of PCOS is crucial for molecular mechanisms underpinning the endoscope hypersecretion feature of PCOS (35). The decrease in the level of fasting insulin recorded by PCOS-treated women with insulin-sensitizing medicine appears to decrease androgenemia while increasing ovarian functionality (36).

On the other hand, whereas this link is generally seen as the one-way highway between IR and hyperandrogenemia, newer studies reveal that IR and hyperinsulinemia may extend. IR and hyperandrogenemia, within the setting of PCOS, can establish a vicious cycle that stimulates each other. This confluence of endocrine and metabolic alterations also provides the basis to further develop the complicating and metabolic therapy of these individuals (37).

## The Possible Causes of PCOS

A mixture of environmental and genetic reasons is causing PCOS. The following causes are highly related to PCOS: excessive embryonic androgen exposure, reactive oxygen species (ROSs), immunological, and endocrine abnormalities (6, 38). At the same time, numerous genes or oligomers seem to cause PCOS. Results of the genetic basis of HA and IR, potential participation of environmental components in PCOS were found in investigations including “family”, “twin babies”, “genome-wide association studies (GWAS)”, genes connected with certain loci, and fetal program (39, 40). Melatonin receptor (MTNR1b) genes all have a relationship with 2PCOS (41, 42) with microRNA expression, SNP rs10830963, and DNA methylation. In patients with PCOS, the blood FSH levels and control gene expression are linked with SLC18A2 genetic variations *in vitro*. In PCOS, the GG allegorice has strong links with the index of body mass (BMI), the hip ratio of waist to hip, the resistance to insulin (IR), luteinizing hormone (LH) and LH/FSH, and a high baseline FSH (43).

PCOS is a non-modifiable risk factor for type 2 diabetes, according to the International Diabetes Federation (44). In the relationship between PCOS and T2D, IR has been established as a common component. Despite the fact that the pathogenesis of IR in PCOS is complex, familial histories of IR, as well as obesity, appeared to be particularly common in afflicted women (45). Aside from that, both the parents’ family and personal histories play a role in the illness being handed down to their children and becoming a family disease. Aside from that, PCOS and T2D share similar traits, hence genetic susceptibility factors have been discovered in both diseases (46). Men and women who are first-degree relatives of PCOS patients have a higher risk of acquiring IR, obesity, and diabetes. It’s unclear whether this has an impact on inheritance methods. IR is well-known for producing pain in PCOS patients who are slim or fat (47). In Virginia hospitals (48), confirmed that PCOS is common in premenopausal women with type 2 diabetes. Pancreatic -cell dysfunction is another risk associated with PCOS and type 2 diabetes (49). T2D-related genes play an important role in PCOS development (50). Positive family histories of PCOS are thought to be a risk factor for PCOS in women. There is evidence that a family history of T2D, as a reflection of genetic risk, is linked to a higher risk of T2D progression in PCOS women; with T2D and obesity-related genes and polymorphisms linked to hyperandrogenism, which has been linked to the PCOS phenotype; implying a significant genetic background (51). Obesity exacerbates PCOS, which is linked to a slew of reproductive, metabolic, and psychological issues, including T2D (52). In 1921 (53), identified beard women with diabetes, and T2D has been linked to PCOS ever since (53). Hyperandrogenism, which is thought to contribute to IR in PCOS and may promote hyperandrogenism, is one probable route. IR does not have to develop in all PCOS women. Obesity is one of the well-established linkages between IR and PCOS, and it will be examined in greater depth in relation to the molecular process. In contrast, the pathophysiology of PCOS differs between obese and non-obese women. In obese people, PCOS is a significant contributor in the development of IR and hyperinsulinemia (54, 55). Women with PCOS may have a higher risk of gestational diabetes (GDM), which is connected to T2D, during pregnancy (56) GDM is a metabolic disorder that affects pregnant women and is defined as carbohydrate intolerance during pregnancy (57). PCOS is described as the presence of small cysts forming in the ovaries, and PCOS and GDM, which was previously referred to as PCOS and T2D, have a similar association. Pan and colleagues (58). Both PCOS and GDM are linked to a higher risk of pregnancy-related hypertension, pre-eclampsia, and infant hypoglycemia. IR, weight gain, and genetic factors were all associated to both of these disorders (59). In reproductive ages, PCOS raises the risk of type 2 diabetes and gestational diabetes mellitus. T2D affects 20% of PCOS women at random, resulting in IGT as a common anomaly (60). PCOS women have an abnormal glucose tolerance, which leads to type 2 diabetes. According to clinical investigations (48), individual family histories of T2D and obesity will increase the prevalence of both diseases in PCOS women; most notably, a family history of obesity greatly contributes to the development of T2D in PCOS women (61).

## PCOS and HA

### Molecular Defects of Hyperandrogenemia

The genetic processes behind polycystic ovarian syndrome (PCOS) and functional hyperandrogenism are mostly unclear. Because of the huge number of genetic variations linked to these disorders, a picture of a complex multigenic trait is emerging, in which environmental factors play a significant role in the hyperandrogenic phenotype’s presentation (62). The challenge in establishing the molecular genetic foundation of these disorders stems from the lack of precision in identifying ethnic and environmental risk factors for hyperandrogenic disorders, as well as the variability of diagnostic criteria used to define PCOS (63). CAG repeats AR and PDE8A polymorphisms, with FST SNP rs 3797297 in PCOS women (25, 31, 32). But the research reveals that CAG microsatellite in the AR gene may not be the fundamental cause of PCOS development frequently polymorphic (64). Intrauterine growth resistances (IUGR), increased androgen exposure, androgen receptors (AR), especially neuron ARs, and poor living conditions include sedentary behavior, longer eating and less training. Hyperandrogenic PCOS (65) causes include PCOS ovarium (66) were discovered to be related to the hypo androgenic nature of the local ovarian immune system PCOS ovaries, but considerable alterations are possible in reactive oxygen, cytokines, and chemical species (67). In PCOS patients, GCs are considered to be inflammatory in genes such IL1B, Interleukin 8, LIF, NOS2, and PCOS2 (52). The leukemia inhibitor GC is composed of interleukin-1beta, IL1B, and interleukin-8 (IL9) (LIF). WNT5a is an inflammatory factor in patients with ovarian grain cells (GCs). WNT5 expression in PCOS increased mainly due to increased inflammation and oxidative stress in the route of the PI3K/AKT-NF-B signal. Expressions of WNT5a can be further stimulated by the NF-B-dependent regulation (68) for pro-inflammatory cytokines generated. PCOS patients in GCs were predominantly hypothesized to contribute to HA following a process of inflammation (69).

### Major Cause of PCOS Is HA

Not merely a sign of clinical PCOS, but HA is the fundamental reason. In utero with high androgen levels, PCOS is reported in fetuses. Prenatal DHT Therapy, comprised of irregular estrogen cycles and progesterone (P4) in a LET-induced mice PCOS model, has discovered several PCOS-related endocrinal anomalies. (Pre-infection androgenization; PNA). Girls with preterm newborns are also susceptible to PCOS, and early visceral and IR secretions prevent. For girls who are born at the start of a small gestational age (SGA), the greatest danger of PCOS is adrenarche (70). In PCOS women’s daughters during childhood, early childhood, and prepuberty, the amount of anti-mullerian hormone (AMH) is higher, the evidence suggests. In addition, the link between the sensitivity of PCOS and the common missense polymorphism enzymatic (rs710059) was established in a study (71). Type I activity was further reduced in PCOS patients using Type I (3-HSD) aromatase (CYP19). In addition, AMH increases in GCs (72), and elevated AMH is combined with insulin resistance/hyperinsulinemia in those with insular induced CYP 19. The comparison between age-specific and lean obesity shows a higher degree of HA in obese patients, which shows negative effects from obesity. However, the metabolic and replica aberrations in PCOS women are constantly improved *via* lifestyle modifications and weight loss (67). The sensitivity and expression of Glu-4 (Glu-4) were established to reduce the degradation of insulin by preventing the liver from degrading and raising central fat that all were important insulin resistance mechanisms. In summary, HA can help to build IR.

## PCOS and Hyperinsulinemia

Important characteristics include insulin resistance (73), elevated blood pressure, dyslipidemia, and central obesity (74), 50-70 percent of PCOS (67). The key characteristics of PCOS and metabolic syndrome are. IR, irrespective of their BMI, is a common feature in PCOS women (75). In obesity, IR typically has a distinctive PCOS adiposity, especially in its central or android form (76). Some PCOS women have a greater phosphorylation-172-1 receptor substratum, which inhibits insulin receptor signal (77). In PCOS, an MTNR1B mutation can delay the synthesis of insulin and produce rapid levels of blood glucose. Insufficient vitamin D can lead to IR in PCOS. Vitamin D encourages the formation of adipose cells, influences lipid and metabolic enzymes by carbohydrate activation, and stimulates tissue breakdown in the adipose (78). Inextricably connected are IR and HA. The excess androgen excess for glucose metabolism sequela and antecedents for future metabolic diseases is significantly higher for newborns exposed to pre-natal androgen (PA). Testosterone may also encourage enlargement of the adipocyte (67). In PA infants the average islet size declined and islets grew proportionally and the fractional area of islets remained constant. Furthermore, the babies showed that the proliferative marker Ki67 was elevated and the cell/+ cell ratio of the islets was increasing (79). Insulin causes theca cells to generate and release androgens direct or indirect (75). Insulin the IR stimulates androgen synthesis in the ovary and lowers the amount of free testosterone (FT) accessible to the body, which inhibits the development of sex hormone-binding globulin (SHBG) in the liver. These results demonstrate that IR can contribute to HA (67). Insulin resistance is a common symptom of PCOS that is unrelated to weight. When compared to weight-matched reproductively normal women, insulin-mediated glucose clearance, which is primarily determined by insulin action on skeletal muscle, is reduced by 35–40% in women with PCOS. 2 Obesity does not cause this insufficiency, but it exacerbates it greatly (80). Hepatic insulin resistance, defined as increased post absorptive glucose synthesis and decreased sensitivity to insulin-mediated inhibition of endogenous glucose production, is only seen in obese women with PCOS when compared to healthy women of equivalent body weight. 2 Obesity and PCOS have a compounding negative effect on endogenous glucose production, which may play a role in the etiology of glucose intolerance (81).

Fasting insulin levels are higher in people with PCOS. There are, however, insulin secretion anomalies that are not linked to fat. Women with PCOS and a first-degree relative with type 2 diabetes are more likely to have these abnormalities. PCOS patients have high basal insulin levels but unusually low carbohydrate insulin responses (8). In normal circumstances, there is a consistent link between insulin secretion and sensitivity, such that changes in insulin sensitivity are matched by reciprocal changes in insulin secretion that maintain normal glucose tolerance; this relationship is known as the “disposition index”. When compared to weight-matched reproductively normal women, women with PCOS, whether obese or not, have a lower disposition index (82) Furthermore, PCOS and obesity have a significant negative impact on the disposition index (83).

### Role of IR in PCOS

Women often experience PCOS (hirsutism, acne, and alopecia), irregulate menstrual cycles, and biochemical alterations associated with elevated testosterone levels, higher dehydroepiandrosterone (DHEA), androstenedione (ASD), reduced SHBG, and the binding protein insulin-related growth factor (IGFBP). These changes are linked to insulin and hyperinsulinemia resistance (66). PCOS is related to insulin and endothelial dysfunction resistance from the start. The levels of oxidative stress in children born to women with PCOS are higher than in pregnant women (84). PCOS female under cutaneous adipose tissue gene expression levels is greater than the general population of CD11c (ITGAX) as well as the alpha tumor necrosis factor (TNF). TNF can aggravate the development of IR in PCOS women as an inflammatory agent. Drops in nitric oxide (NO) and higher endothelin 1 levels (ET-1) result in IR in endothelial artery cells. At the same time, vasoconstrictors are produced and vasodilation induced by insulin is reduced. Therefore, IR raises the risk of cardio visual and metabolic illnesses for PCOS-positive women, according to the American Heart Association (27). IR raises the likelihood of getting type II diabetes. The release of insulin from pancreatic cells is increased as a result of IR in PCOS women. Hepatic production is elevated in IR and adipose tissue mobilized which leads to increasing levels of plasma-free fatty acid (FFA). Increased FFA contributes to IR by inactivating major enzymes, including glucose transport functions, such as dehydrogenase pyruvate (PDH). The process involves an insulin signaling sequence that influences PI3 kinase-1 (IRS-1) receptor substratum decrease, according to specialists. This means that both hepatic glucose production and insulin inhibition are enhanced. The liver function is also changed and complies with IR (66).

Study shows that after 12 and 24 weeks of therapy, all individuals had significantly lower plasma insulin levels (from 14.2 ± 1.1 to 11.7 ± 0.9 and 9.10 ± .8 μU/ml, p<0.004, p<0.03). Triglyceride, total cholesterol, and the Homeostatic model assessment (HOMA) index all fell considerably, but high-density lipoprotein rose significantly. Of the 45 PCOS patients 39 had a hyperinsulinemic response during oral glucose tolerance tests. All metabolic indices and the hepatic insulin extraction index (HIE) were significantly reduced in this group. In normoinsulinemic PCOS individuals, no alterations were detected (6 out of 45) (85).

Patients with hyperinsulinemic PCOS had the most severe metabolic abnormalities. A combination of nutraceutical substances, including acetyl-L-carnitine, L-carnitine, L-arginine, and N-acetyl cysteine, significantly improved metabolic indices and the HIE in overweight/obese PCOS individuals, particularly hyperinsulinemic subjects. The improvement in HIE supports the theory that liver function is compromised in hyperinsulinemic PCOS.

## Mutual Act of IR and HA on Ovaries and Adrenal Glands

HA is mostly due to dysregulated steroid biology in women who have PCOS that is to say to an imbalance in the function of the adrenal cortex and of the ovary (86). The prevalence for women with PCOS varies between 15% and 45% in adrenal hyperandrogenism. The sulfotransferase function of sulfotransferase 2A1 (SULT2A1) (87) mainly converts DHEA into DHEAS in the suprarenal cortex. The adrenal gland produces the majority of DHEAS that circulates due to its poor expression in ovarian tissues. DHEA is the most abundant human precursor to steroids, and up to 97% of circulating DHEA is produced by the adrenal gland. In patients with conventional anovulatory PCOS, DHEAS levels increased substantially (67). However, and possibly especially, because of their diurnal variation, intersubject variability, and heightened stress, the diagnostic utility of DHEA in PCOS has been constrained. Thus, elevated DHEAS shows an over-production of androgen by the suprarenal glands. In certain PCOS women, HA results in the creation of dysfunctional adrenal steroids (functional adrenal androgen excess [FAH]). The production of androgen in the adrenal reticular band is controlled by adrenocortical hormones (ACTH). The Hyperactivity of Adrenaline to ACTH is a characteristic of PCOS and AH symptoms. Although polymorphisms of the 11-hydroxysteroid dehydrogenase gene (HSD12B1) are not connected to PCOS (88), in the case of HSD11B1 liver reliant peripheral cortisol production it could result in compensatory HPA axis stimulation. Ovarian testosterone may also impair liver enzyme activity, which causes deleterious consequences. Regenerating cortisol is an important source of cortisol in the suprarenal system. Insulin, however, may boost HSD11B1 activity in adipocytes using the P38 Signal Protein kinase (MAPK) pathway. Hyperactivity in the HPA axis may also be associated with the accelerated peripheral cortisol clearance for hepatic 5 in women with PCOS as a consequence of IR (89). This can be explained by IR/hyperinsulinemia. The compensatory HPA axis is a fascinating approach for PCOS patients to comprehend AH. One reason could be an AH in PCOS Induced by an acquired mechanism such as insulin resistance/hyperinsulinemic, P450c17 was an increase in 5-17 hydroxylase (CYP17) and/or 5-17.20 lysis activities. However, in a study that looked at specific PCOS quantitative traits, no connection was identified between CYP17 genes and typical PCOS quantitative traits. The 5-P450c17 regulates post-translation pathways, including the lack of serine kinase activity in PCOS patient’s results in an increase in cortisol (including aldosterone) and insulin resistance (IR). Short-term infusions of high insulin doses increased in PCOS women, while metformin and pioglitazone lowered 17OHP and ASD when induced by ACTH. Deficiencies like these may promote or contribute to obesity-related diseases such as hyperinsulinemia and/or other metabolic issues. Additionally, adrenal reticular cells synthesize DHEA from Pregnenolone, which is derived from adrenocorticotrophic hormone (ACTH) stimulation, due to the poor expression of type II 3-hydroxysteroid dehydrogenase (HSD2) (90). Cortisol is the primary synthesis of cortisol. This ratio, of 5 females for every 4 men, went raised in PCOS, due to the partial inactivity of 3-hydroxysteroid dehydrogenase (3-HSD). This statement agrees with IR (91) and is a viable candidate for AH in PCOS, considering PCOS itself is an underlying condition for P450c17 and 3-HSD regulation alongside the P450c17/3-HSD regulation with ERK/MEK signaling pathways. Additionally, due to higher quantities of DHEAS in the blood, AH’s popularity may be racially biased. It has been claimed that T’s production of AH begins at rest and then increases in response to ACTH. The results indicated that testosterone injection increases DHEA levels in the NCI-H295R human adrenocortical cell line and that it also reduces DHEAS levels. T was also present in newish adrenal tissue taken from typical women, but in the adrenal glands of these ordinary women, T did not affect the quantity of DHEA or DHEAS (89). Additionally, ovarian steroids have a direct impact on adrenal steroid synthesis. Additional research on production is needed. In persons with PCOS, metformin was reported to reduce IR (also known as insulin resistance) as one of the insulin sensitizers. We would like to notice that. In women with glucose-mediated or rapid insulin levels, metformin medication has been shown to decrease PCOS concentrations. While metformin use was associated with decreased fetal insulin concentrations among PCOS women, it did not impact fetal insulin concentrations in PCOS women who were taking metformin (92).

**Figure 1:**
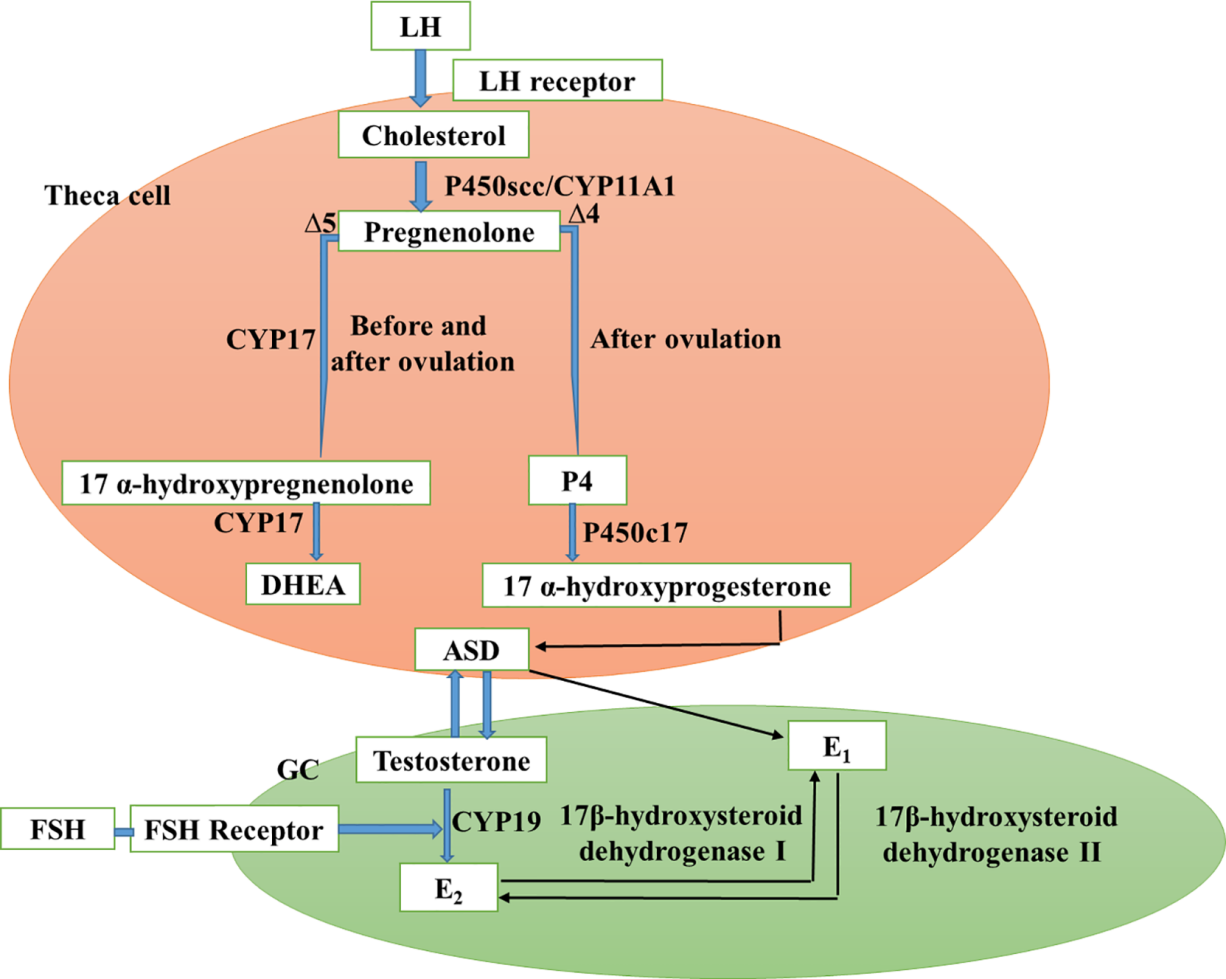
Schematic representation of the PCOS ovarian hormones (Adapted and modified from (97).

The level of insulin is lower in pregnant women (93). However, lower insulin activity can be different from that for women with type II diabetes or obesity in women with PCOS. The fact that metformin promotes insulin secretion, especially in the early stages of secretion, is unacceptable without the traditional symptoms of PCOS (94). More research on metformin’s influence on insulin levels is thus justified. Interestingly, metformin drugs, perhaps due to reduced glucose concentrations, were demonstrated to enhance HA. In PCOS (95, 96), levels of insulin are elevated. That also means that HA and IR are very strongly linked. **Figure 1** demonstrates a schematic representation of this PCOS hormone-releasing. In PCOS, the HPO axis is out of whack. GCs first carry out this function during the development of the sinus follicles, while follicular theca and follicular GCs both play a critical role in the synthesis of steroid hormones. GCs (Gastrulation Conditioned Squamous Epithelial Cells) are first activated by FSH and subsequently LH during the monthly cycle, whereas follicular cells (Follicle Cells) only respond to LH. This two-cell, two-gonadotropin (FSH, LH, and androgens) theory explains the production of androgens from androgen precursors with follicular theca, GCs, and LH, as well as FSH. To convert cholesterol to androgens, three steroid enzymes (CYP11A, 3-HSD, and CYS17) are expressed in the ovarian membrane cells. The CYP11A (or P450scc) mitochondrial enzyme clicks the cholesterol side-chain and subsequently creates Pregnenolone that spreads swiftly from mitochondria. Once converted to pregnenolone, 17-hydroxy pregnenolone or P4 can be metabolized further by CYP17 or 3-HSD, respectively. Converting 17-hydroxypregnenolone to DHEA is catalyzed by the CYP17 enzyme. ASD is a 3-HSD precursor to ASD; ASS is the major precursor of follicular theca cells released by dihydrotestosterone and testosterone. GCs absorb ASD. ASD (98, 99).

In the presence of FSH and *via* a basal layer, CYP19 transforms testosterone into estradiol. The major estrogen is estradiol (67). Estradiol (E2) is then converted by a chemical process into estrone (E1). 17-hydroxysteroid dehydrogenase type I is converted to estradiol by 17-hydroxysteroid type 2 dehydrogenase. The primary steroid 317 routes are arranged and regulated by the hypothesis of two-cell-two-gonadotropin biosynthetic theory in the small Antral follicle of PCOS. Due to increased frequency of the release of GnRH by the hypothalamus, increased GnRH sensitivity, and excessive insulin on hypophysial insulin receptors, excess LH is produced in PCOS patients, thus encouraging the production of excessive androgen by ovarian stroma and follicular membrane cells (100). In between, androgen releases from the suprarenal gland can be enhanced by IR, SHBG synthesis limited, and free testosterone increased. The high levels of androgen in the ovary contribute to the inhibition and prevention of follicles in follicles. However, at the early follicle level, the small follicles in the ovary can continue releasing E2. In addition, androstenedione is metabolized by CYP19 to E1, leading to increased levels of estrone in peripheral tissues. Continuous secretion of E1 and certain E2 levels on the pituitary and hypothalamic secretion does not produce an LH-pick-pick with the center of menstruation, increase the amplitude and frequency of LH Secretion, prove to be continuously high with no periodicity (101). Estrogen also functions as a negative FSH feedback mechanism, decreases the FSH level, and increases the LH/FSH ratio. When the high level of LH is present, the ovary is activated and androgens are produced, but the low level of FSH prevents follicle growth, creating a vicious cycle of hyperandrogenism and anovulation. This allows for the development of polycystic abnormalities in the ovary (102). Abbreviations: CGs: granular cell; LH: Hormones luteinize; FSH: hormone follicle-stimulating; DHEA: dehydroepiandrosterone; P4: progesterone; ASD: Androstenedione; E2: estradiol.

PCOS has a distinct neuroendocrine phänotype defined under the effect of IR, which improves the overall synthesis of LH and FSH production *via* sustained, fast GnRH pulsation. The LH/FSH ratio is thus increased (103). Increased CYP11A1 and CYP17 expression may lead to an increase in the production of androgen in women suffering from PCOS (89). The LH-dependent character of ovarian hypertension may be useful to explain why PCOS usually occurs as puberty reactivates the reproductive hypothalamus-hospital axis and increases LH secretion (104). In regulating the occurrence of HA in PCOS thus, IR is crucial. The usual hallmark of PCOS is excess testosterone of follicular origin. Tissue and GCs treated with androgen have been observed to induce circadian rhythms. Distributor-dependent phase-dependent activities. In androgen-treated mice, estrous cycles were stopped. Flutamide treatment nevertheless can restore the estrous cycle in PCOS animals, lower ovarian-like follicles in LET females, and reduce a variety of people’s PCOS symptoms, including P4 reactivity. Loss of signals from androgen receptors (AR) improves the PCOS model phenotype. Excess androgen may thereby modify the hypothalamic-hypophytic-gonadal axis by AR, which reduces the susceptibility of P4 to negativity. This leads to neuroendocrine dysfunction in the PCOS (103) that undermines ovarian function.

## Reproductive Failure Due to IR and HA in PCOS

Reproductive abnormalities that present as infertility (75 percent of anovulatory infertility is PCOS) and an increased risk of abortion (105, 106) are the most important concern in PCOS patients with childbearing age. Anomalies of ovulation are induced by faulty endocrine metabolism, reduced oval formation capability, and reduced endometrial receptiveness (ER). The ovary, in which HA and IR may alter ovarian follicle growth and also fertile oocyte formation, is the principal organ affected (100). Anovulatory phenotype PCOS is more probable than typical PCOS to have IR. Dominant follicular GCs create large IGF-II volumes during the follicular phase in the follicular fluid. The levels of the IGF-II in folic fluid have a positive correlation with the diameter of the follicle and E2, but with the androgen. Non-dominant follicles have low IGF-II levels and this effect is not magnified, causing developing follicle defects. HA leads to reduced levels of IGF-II in follicular fluid in women with PCOS. The follicular theca cell death can be inhibited by estrogen produced by many follicles that cause sinus follicles stagnation, not obstruction in PCOS. In women with PCOS, more LH encourages the development of ovarian theca cell androgens, whereas inadequate FSH helps impaired folliculogenesis and anovulation. Insulin resistance/hyperinsulinemia in women with PCOS encourages androgen synthesis directly in ovarian and ductless glands, increasing follicular maturation and leading to anovulatory infertility. Intriguingly, IR/hyperinsulinemia promotes pituitary LH release, boosting androgenic production and inhibiting SHBG synthesis, resulting in high levels of FT (66). This disrupts both ovarian and ovulatory functions. Ovulatory dysfunction (75) is most typically caused by infertility. PCOS can lead to ovarian failure in mutations in the gene of LH chorionic gonadotropin (LHCGR) receptor. Exotropinism may also lead to ovarian collagen fibrosis, which results in abnormal tunic thickness, which makes follicles less susceptible to rupture, leads to un-ruptured follicle luteinized (LUFS) syndrome, also linked to infertility. Insufficiency in vitamin D has been associated with poor outcomes in PCOS stimulation (107). The deficiency of vitamin D3 (VitD3) leads to the normalization of serum AMH and promotes follicles (101). That means, in the development of PCOS oocytes, vitamin D plays a key part. HA, IR and higher LH levels are generally strongly affected by the production of ovary follicles and could lead to anovulatory cycles (66).

## IR and Ageing

Both sexes have a continuous increase in body weight with the advancement of age, which is associated with a detrimental effect on metabolic profile, and IR has long been considered the primary pathophysiological link between obesity and metabolic abnormalities (108, 109). Additionally, ageing is associated with a steady increase in IR and -cell decompensation in a healthy population, which results in the development of diabetic mellitus (DM) (110). Nonetheless, the molecular mechanisms behind IR in persons with DM are distinct from those underlying IR in people with PCOS, and patients with DM exhibit varying degrees of IR in various organs (111).

According to study, women with PCOS had a higher level of intrinsic IR than their age- and BMI-matched contemporaries (112, 113). Women with PCOS also had higher HOMA-IR readings than women without PCOS, independent of BMI (114). As a result, the notion that PCOS is a risk factor for the development of diabetes in non-obese women with the syndrome should be re-examined, particularly given that the current findings come from a cross-sectional rather than a prospective investigation.

It has been clear over the last two decades that both IR and -cell dysfunction is necessary for the development of diabetes mellitus, and that both of these illnesses are associated with ageing (109, 115). If, on the other hand, IR improves with time in non-obese PCOS women, this trait can compensate for the decreased -cell secretion, hence lowering the risk of diabetes. Additionally, while thin women with PCOS have intrinsic IR, the degree of IR is comparable to that of their obese control peers (8, 116). As a result, obesity appears to be a substantial risk factor for the development of IR, and one may argue that DM in women with PCOS is an epiphenomenon caused by an elevated BMI, given the common coexistence of obesity and PCOS. This concept was advanced by the Escobar-Morreale group, which discovered that overweight and obese women have a significantly higher prevalence of PCOS than lean women (28.3 vs. 5.5 percent, respectively), a finding validated by other study groups (117, 118). Additionally, women with PCOS have a high familial history of diabetes, which is another significant risk factor for diabetes development (119).

The steady fall in IR throughout time may be a result of the natural decline in androgen levels associated with ageing. In PCOS, IR and hyperandrogenemia are mutually exclusive, and numerous *in vitro* and *in vivo* investigations have indicated that reducing androgen levels improves IR (120). Additionally, a direct correlation between testosterone levels and the risk of developing IR or DM has been established in pre- or postmenopausal normal women (121). Androgen levels continuously decline with time in women with PCOS and controls, as previously demonstrated (122) and corroborated in this study. Androgens, on the other hand, declined independent of BMI, demonstrating that the relationship between androgens and age is direct and not indirect *via* fat (123).

## Current Clinical Treatment of PCOS

It is difficult to produce a PCOS-specific medication (124) due to its complexity and range of female clinical characteristics. The majority of treatment regimens advise PCOS women to change their lifestyles, such as exercise, diet, and weight loss. The treatment in the first line of PCOS menstrual problems and hirsutism/acne for women with PCOS can be used as oral contraceptives (OCPs). The usage of androgen-excessive behavior is anti-androgens. Medicines that sensitize insulin can be used to treat low glucose tolerance or symptoms of metabolic illness. Anovulatory infertility is treated using clomiphene citrate or related estrogen modulators such as letrozole (LET) in women with polycystic ovarian syndrome (PCOS) (125). The surgical interventions are laparoscopic ovarian perforation (LOD) and ovarian wedge resection (126). Patients with PCOS should have their treatment progress modified to meet the treatment goals of patients and doctors, as there are no single treatments now (127). Women with PCOS should consider lifestyle changes first, such as food re-calibration and increased physical activity [(128); Consensus on infertility treatment related to polycystic ovary syndrome], especially if their BMI is greater than 25 kg/m2. To enhance fertility, 343 obese infertile women with PCOS were randomly assigned to receive clomiphene citrate alone, metformin alone, a combination of the two, or a lifestyle change program (low-calorie diet and risk-free activity for 30 minutes per day) (129). Women in the lifestyle group outperformed those in the pharmaceutical group in terms of waist circumference, LDL cholesterol, and insulin levels, although SHBG levels improved similarly in both groups. More crucially, despite the fact that the difference was not statistically significant, the pregnancy rate in the lifestyle group (20%) was significantly greater than in the combo group (14.8%). 30 obese, insulin-resistant PCOS women were randomly assigned to lifestyle modification plus metformin or a placebo for four months in a recent clinical trial (130). The researchers discovered that a small weight loss achieved through lifestyle adjustments was sufficient to alter PCOS patients’ menstrual cycles, and that metformin had additive effects on insulin resistance and hyperandrogenism. In obese PCOS women, weight loss of just 5% of their starting body weight can result in conception (131), whereas weight loss of 5–10% can lower hyperandrogenism and insulin levels (132).

There is currently no credible evidence on which meal composition is optimal for improving PCOS clinical results. For 12 weeks, 28 overweight PCOS women were randomly assigned to either a low-protein or high-protein diet (133). Although there was no significant difference in food content, both diets reduced body weight (7.5%) and belly fat (12.5%), as well as improved pregnancy rates, menstrual cyclicity, lipid profile, and insulin resistance. Weight reduction, clinical, and biochemical changes were not statistically significant in a randomized controlled experiment comparing high-protein and high-carbohydrate diets (134). If fatty acid buildup in androgen-secreting cells is linked to PCOS pathogenesis, the fat content of the diet may become more important than the other macronutrients. Saturated fatty acids, for example, were found to concentrate in cells and enhance testosterone levels in male rats to a greater extent than polyunsaturated fatty acids (PUFA) but to a lesser amount than monounsaturated fatty acids (MUFA) (135). Supplementing with PUFAs for an additional three months after a three-month normal diet improved glucose homeostasis, plasma lipids, and sex hormones in women with PCOS, according to a prospective study (136). According to a cross-over trial comparing eucaloric diets higher in MUFA to those low in carbs, the low CHO diet had a lower acute insulin response to glucose than the MUFA diet (CHO). Diets were only examined for 16 days, which is insufficient time for fat modulation to influence insulin sensitivity and testosterone levels. Given the scarcity of publications evaluating the significance of dietary fat content in women with PCOS, we propose that more research be done to better characterize and understand the impact of dietary fat on PCOS management.

After non-pharmacological approaches fail, medications for insulin-related hyperandrogenism and insulin resistance can be recommended to women with PCOS. Metformin, thiazolidinediones (TZDs, PPAR agonists), D-chiro- or myo-inositols, and acarbose, among other insulin-sensitizing or insulin-lowering medications, have been demonstrated to diminish hyperandrogenemia in both lean and obese women with PCOS (137). Metformin is a biguanide that lowers hepatic glucose synthesis while improving insulin sensitivity slightly. Furthermore, this medication reduces hunger in a substantial percentage of PCOS women, and is thus frequently (138), but not always (139), associated with weight loss. Metformin has been proven to help all women with PCOS, including those without insulin resistance or hyperinsulinemia (140), but it is more helpful in lean PCOS women than obese PCOS women (141). Metformin’s benefits on PCOS are most likely mediated by a reduction in insulin levels, which can be seen in both insulin-sensitive and insulin-resistant PCOS women due to a decrease in hepatic glucose production. Metformin appears to directly inhibit androgen synthesis on the ovaries (142), which could be linked to an increase in intracellular FFA buildup. This hypothesis, on the other hand, needs to be tested *in vitro*.

TZDs are another type of insulin-sensitizing drug that can be used to treat PCOS symptoms. In adipocytes and androgen-secreting cells, TZDs increase gene transcription and activate genes that code for insulin action and proper FFA metabolism. TZDs, unlike metformin, are real sensitizers that help people with normal insulin sensitivity maintain their insulin levels. Troglitazone, rosiglitazone, and pioglitazone have all been approved by the FDA; however, troglitazone has been discontinued due to idiosyncratic hepatotoxicity. Several studies (143, 144) have found that using one or more TZDs can benefit women with PCOS with insulin resistance, ovarian dysfunction, and hyperandrogenism. TZDs like metformin have been shown to improve hyperandrogenism and ovulation rates in slim women with PCOS and normal insulin levels (145). TZDs appear to be at least as effective as metformin in treating the clinical symptoms of PCOS (127). For example, in obese PCOS patients treated for 12 weeks with metformin, orlistat (a weight loss inducer), or pioglitazone, all three medications effectively reduced hyperandrogenemia characteristics (146).

The adrenal fasciculata and ovarian thecal cells both have PPAR receptors, and their ligands have been demonstrated to lower P450c17 and 3HSD2 activity in human adrenal cells while enhancing testosterone synthesis in pig thecal and human ovarian cells (147). Furthermore, PPAR agonists have been found in human adrenal cells to reverse the increased expression of P450c17 produced by MEK/ERK suppression (148). As a result, PPAR appears to play a direct role in androgen synthesis, suggesting that activating this receptor could assist to ameliorate some of the insulin signaling protein anomalies connected to PCOS hyperandrogenemia. Furthermore, because all insulin-sensitizing medications improve adipocyte insulin sensitivity, it’s possible that this is a common mechanism by which insulin sensitization relieves hyperandrogenism.

Another line of treatment includes laparoscopic surgeries; this technique is carried out under video surveillance in the lithotomy position (149). As a result of developments in minimally invasive surgery technology, laparoscopic surgeries that need fewer port wounds, single incisions, or use of the natural orifice have gained in favor (150, 151). As a result, the single-port laparoscopic approach can also be used to execute LOD. The conventional three-port wounds for LOD are summarized below. A 5–10 mm trocar is placed in the umbilical position, and two 5 mm trocars are placed in the right and left lower quadrants, 6–8 cm lateral to the inferior epigastric artery and oblique to the pubic rami, to position the video scope. To grip the utero-ovarian ligament and move the ovary away from the intestine and ureter, a set of grasping forceps is inserted *via* one of the 5 mm trocars. On a single ovary or both ovaries, three to ten diathermic punctures (each 3 mm in diameter and 2–4 mm in depth) are commonly conducted utilizing 600–800 joules (J) of energy. However, because the clinical effects of LOD may be dose-dependent, it is advised that each ovary receive at least 600 J, as recommended by (152) in their initial study on the amount of energy utilized for LOD. The duration of each penetration is between 2 and 4 seconds. After chilling the bilateral ovaries with an isotonic solution, the existence of bleeding is detected. Finally, 500–1000 mL of normal saline should be injected into the cul-de-sac to cool the ovaries and avoid heat harm to nearby tissues, as well as to lower the chance of postoperative adhesion formation and effectively treat postoperative shoulder tip pain (153, 154). In order to maximize therapy response with the least amount of follicle injury possible, the best amount of electrosurgical energy to utilize at each puncture is uncertain (155). compared the effects of LOD on metabolic consequences using two distinct cautery procedures. In group A, four 5 s or five 4 s punctures were employed with a voltage (V) of 3040 to obtain a total energy of 600 J per ovary. Group B’s energy measurement (based on ovarian volume) was based on earlier research that employed 640, 450, 600, and 800 J per ovary (mean: 625 J). There were no significant variations in AMH, testosterone, or dehydroepiandrosterone sulphate (DHEA-S) levels between the two groups, according to the researchers. Additional LOD procedures are required, such as office micro laparoscopic ovarian drilling (OMLOD) performed under augmented local anesthetic rather than general anesthesia (156). OMLOD has a number of advantages, including a faster recovery period, less pain, and less hospitalization. Fertiloscopy (transvaginal hydro laparoscopy) has also been described as a viable ovarian drilling approach (157). LOD has also been proposed using a harmonic scalpel and a monopolar hook electrode (158).

## Conclusion and Future Perspectives

PCOS is an extremely complex illness with several phenotypes that sometimes makes it difficult to recognize and treat. Therefore, many groups around the world developed criteria of consensus. This article describes PCOS-based physiopathology, which is linked to HA and/or IR-mediated symptoms. PCOS androgenism has a convoluted etiology closely linked to the ovaries and suprarenal. The development of systemic diseases in PCOS can be influenced by HA and IR. They are intricately connected to breeding processes, obesity, hypertension, NAFLD, dyslipidemia sleep, neuroendocrine issues, apnea, AGEs, and EDC impacts. PCOS is usually identified by irregular menstruation or infertility in young women; PCOS can in its later phases create a range of metabolic problems. Cognitive and behavioral pathways are likely to be involved, as in women of PCOS, in part because of their distressing symptoms; anguish and despair, smoking and excessive alcohol use and inactivity are widespread. Timely training and interventions to improve one’s quality of life. Interactions with variables such as weight and food are increasingly recognized as having the potential to change the nature of PCOS. More studies are also needed to link the underlying causes of PCOS (HA and IR) with clinical events and to develop more scientifically and clinically relevant therapeutic approaches

## Author Contributions

HD; data collection and manuscript write, JZ; Data analysis and Manuscript write up, FZ; study design and data interpretation, SZ; Manuscript write up, XC; study design and data interpretation, WQL; study and Supervised the manuscript, QX; Reviewed the manuscript and edited.

## Funding

This work was supported by the Nature Science Foundation of Zhejiang Province (LY18H060013 to WQL), Foundation of Zhejiang Province medical health (2015PYA012, and 2016RCA029 to WQL).

## Conflict of Interest

The authors declare that the research was conducted in the absence of any commercial or financial relationships that could be construed as a potential conflict of interest.

## Publisher’s Note

All claims expressed in this article are solely those of the authors and do not necessarily represent those of their affiliated organizations, or those of the publisher, the editors and the reviewers. Any product that may be evaluated in this article, or claim that may be made by its manufacturer, is not guaranteed or endorsed by the publisher.
